# A Case of HER2 Mutated Colorectal Cancer Treated Successfully With Fam-Trastuzumab Deruxtecan

**DOI:** 10.7759/cureus.38582

**Published:** 2023-05-05

**Authors:** Aswanth Reddy, Nkolika Nwankwo, Arjun Sekar, Aswini Kumar

**Affiliations:** 1 Hematology and Oncology, Mercy Clinic, Fort Smith, USA; 2 Medicine, Mercy Clinic, Fort Smith, USA; 3 Nephrology, Rochester Regional Health, Rochester, USA; 4 Cardiology, Mercy Clinic, Fort Smith, USA

**Keywords:** human epidermal growth factor receptor 2 (her2), next generation sequencing (ngs), anti-her2 therapy, trastuzumab-dexuxtecan, colorectal cancer

## Abstract

Colorectal cancer is a malignant tumor arising from the inner lining of the colon or rectum and is the third most common cancer and the third leading cause of cancer-related deaths in the United States. Human epidermal growth factor receptor 2 (*HER2) *gene* *overexpressed or amplified colorectal cancer has shown treatment responses with *HER2*-directed therapies. We present a 78-year-old woman with metastatic colorectal cancer with a *HER2 L726I* mutation identified in tumor sequencing without amplification or overexpression of *HER2*. She had an excellent response to fam-trastuzumab deruxtecan. Our case is the first and most noteworthy case of a patient with metastatic colorectal cancer and a *HER2 L726I* mutation who achieved a remarkable clinical response to fam-trastuzumab deruxtecan.

## Introduction

Colorectal cancer (CRC) is a malignant tumor arising from the inner lining of the colon or rectum and is the third most common cancer and the third leading cause of cancer-related deaths in the United States [1]. The traditional adenoma-carcinoma pathway (which leads to 70-90% of colorectal cancers) and the serrated neoplasia pathway are attributed pathways of cancer formation from a polyp [2]. Although surgery continues to be a primary modality of treatment for non-metastatic rectal cancers, a total neoadjuvant approach with chemotherapy and radiation has been widely used in recent times [3]. Advanced or metastatic colorectal cancers are treated primarily with chemotherapy-based regimens in the first-line setting. Targeted therapies such as anti-EGFR (epidermal growth factor receptor), anti-VEGF (vascular endothelial growth factor) agents, and immunotherapy are used in second-line settings in patients expressing specific biomarkers [4]. *HER2* (human epidermal growth factor receptor 2) is a proto-oncogene that does not bind ligands but dimerizes with other *HER2* receptors and induces the downstream activation of signal transduction pathways [5]. *HER2* overexpressed or amplified colorectal cancer has shown treatment responses with HER2-directed therapies. We present a HER2 mutated colorectal cancer case without *HER2* amplification responding significantly to fam-trastuzumab deruxtecan.

## Case presentation

**Figure 1:**
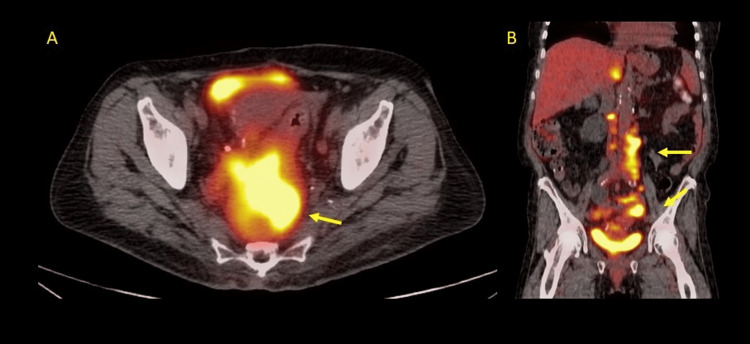
PET scan images Positron emission tomography (PET) scan showing a large presacral mass (arrow) in panel A and retroperitoneal and pelvic lymphadenopathy (arrows) in panel B.

We present the case of a 78-year-old Caucasian woman with a history of rectal adenocarcinoma in 2017. She underwent low anterior resection, with final pathology revealing stage IIIA (T1, N1b, cM0 American Joint Committee on Cancer {AJCC} 7th edition). Despite declining recommended adjuvant therapy, she was monitored clinically with active surveillance. In 2019, she had an incidental right kidney mass that was successfully resected by partial nephrectomy. Final pathology showed clear cell subtype renal cell cancer. In September 2022, she was admitted to the hospital with symptoms of bowel obstruction. Imaging studies revealed a complex solid and cystic mass in the right pelvis, retroperitoneal lymphadenopathy, pelvic lymphadenopathy, right hydronephrosis, and multiple non-calcified pulmonary nodules. Surgical exploration with diverting colostomy and biopsy confirmed metastatic adenocarcinoma consistent with the colorectal primary. Immunohistochemical analysis of the tumor revealed positive CK20, CDX2, and negative CK7 and PAX8 stains. Positron emission tomography (PET) confirmed multiple sites of metastatic disease, including bilateral lung nodules, retroperitoneal lymphadenopathy, and a large presacral mass (Figure 1).

**Figure 2:**
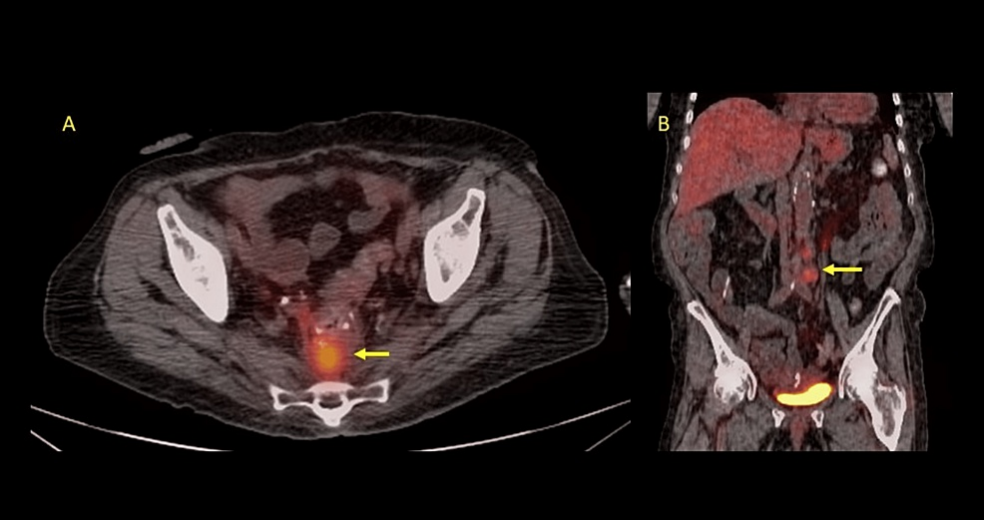
PET scan images 12 weeks after treatment The images show near complete resolution of presacral mass (arrow) in panel A and significant improvement in retroperitoneal lymphadenopathy (arrow) in panel B.

Chemotherapy was recommended, but the patient declined due to concerns about potential side effects. Next-generation sequencing on the tumor specimen revealed mutations in the *ATM* (ataxia-telangiesctasia mutated) and *HER2 (ERRB2 L726L)* genes, with a tumor mutational burden of 7.9 m/MB. Germline genetic testing was negative for pathogenic mutations, and immunohistochemistry and fluorescence in situ hybridization (FISH) testing for *HER2* expression on the tumor specimen were negative. Despite the negative *HER2* amplification, treatment with fam-trastuzumab deruxtecan was initiated after obtaining the drug for compassionate use from the drug manufacturer. The patient tolerated the treatment well, with the only significant side effect being diarrhea (grade 2 by Common Terminology Criteria for Adverse Events v5.0), which was managed with dose reduction and an antidiarrheal medication. A repeat PET scan after 12 weeks (about three months) of treatment showed significant improvement in all known disease sites (Figure 2) and a significant improvement in the carcinoembryonic antigen tumor marker from 154 ng/mL to 3.5 ng/mL.

## Discussion

*HER2* amplification represents approximately 2% of all stage 4 colorectal cancers and is associated with resistance to *EGFR-*based treatment [6,7].* HER2* overexpression is defined >=50% staining by immunohistochemistry (IHC) or >=10 % staining by IHC and positive amplification by fluorescent in situ hybridization as per HERACLES Diagnostic Criteria [8]. Amplification or overexpression of *HER2* oncogene causes hyperactivation of mitogenic signals, even without ligand binding, thereby leading to uncontrolled cell proliferation and tumorigenesis [9]. With next-generation sequencing, several *HER2* gene mutations are being identified, and the concordance of *HER2* mutation with* HER2* overexpression/amplification is seen in about 82% of patients [10]. Preclinical studies using single-agent *HER2*-directed drugs did not show promising results; hence the clinical trials aimed at studying combining chemotherapy and *HER2*-directed drug or a combination of dual *HER2* blockade. HERACLES and MyPathway are phase II trials that studied dual *HER2* blockade in metastatic colorectal cancer. HERACLES trial reported a median progression-free survival (PFS) of 4.7 months and 4.1 months in cohort A (cohort A: trastuzumab plus lapatinib) and cohort B (cohort B: pertuzumab and trastuzumab emtansine) respectively [11,12]. A combination of trastuzumab and pertuzumab was studied in a phase II trial (TAPUR study) and reported a disease control rate (DCR) of 54 % and an objective response rate (ORR) of 25% in *HER2* amplified patients. However, no ORR was observed in *HER2*-mutated patients [13].

Trastuzumab deruxtecan was studied in DESTINY-CRC01, a phase 2 trial that treated *HER2-* expressing metastatic colorectal cancer and resulted in an ORR of 45.3% in 86 treated patients [14]. Recently FDA (Food and Drug Administration) approved the first *HER2*-based treatment, Tucatinib, for metastatic colorectal cancer in combination with trastuzumab [15]. Tucatinib with trastuzumab was studied in the MOUNTAINEER trial, and the investigators reported a 38.1% ORR and a DCR of 71.4%. The data we reviewed shows several options for treating *HER2* overexpressed or amplified colorectal patients beyond the standard of care treatment options. Unfortunately, no promising results were observed in patients with *HER2* mutated but not overexpressed or amplified.

Our review identified a patient's case where the *HER2 S310F* mutation achieved clinical response to a combination of trastuzumab + lapatinib and trastuzumab deruxtecan [16]. Interestingly this patient also had *HER2* amplification. Our patient's *HER2 (ERBB2 L726I)* mutation has never been reported in patients with colorectal cancer. *L726I* mutation has been shown to result in resistance to gefitinib in a preclinical cell line study [17] which raises a question on the significance of *EGFR* therapy resistance (cetuximab or panitumumab) in colorectal cancer patients with* L726I* mutation like our patient.

## Conclusions

This report presents a first and noteworthy case of a patient with metastatic colorectal cancer and a *HER2 L726I* mutation who achieved a remarkable clinical response to fam-trastuzumab deruxtecan. The significance of this case lies in emphasizing the importance of comprehensive genomic testing in the context of metastatic cancers. Our case highlights the importance of conducting additional trials to determine the suitable treatment for patients with uncommon mutations, similar to what we observed in our patient's case.
