# Correction: A Case of HER2 Mutated Colorectal Cancer Treated Successfully With Fam-Trastuzumab Deruxtecan

**DOI:** 10.7759/cureus.c124

**Published:** 2023-06-16

**Authors:** Aswanth Reddy, Nkolika Nwankwo, Arjun Sekar, Aswini Kumar

**Affiliations:** 1 Hematology and Oncology, Mercy Clinic, Fort Smith, USA; 2 Medicine, Mercy Clinic, Fort Smith, USA; 3 Nephrology, Rochester Regional Health, Rochester, USA; 4 Cardiology, Mercy Clinic, Fort Smith, USA

This article has been corrected at the request of the authors to correct the following sentence in the abstract: “We present a 78-year-old woman with metastatic colorectal cancer with a HER2 L726I mutation identified in tumor sequencing **with** amplification or overexpression of HER2” has been changed to “We present a 78-year-old woman with metastatic colorectal cancer with a HER2 L726I mutation identified in tumor sequencing **without** amplification or overexpression of HER2”.

The authors and journal deeply regret that this error was not identified and addressed prior to publication.

 

